# Knowledge, attitude and preventive practice towards tuberculosis among clients visiting public health facilities

**DOI:** 10.1186/s13104-019-4292-2

**Published:** 2019-05-15

**Authors:** Ayele Semachew Kasa, Alebachew Minibel, Getasew Mulat Bantie

**Affiliations:** 1 Department of Adult Health Nursing, College of Medicine & Health Sciences, Bahir Dar University, Bahir Dar, Ethiopia; 2 GAMBY Medical and Business College, Public Health Department, Bahir Dar, Ethiopia

**Keywords:** Tuberculosis, Knowledge, Attitude, Practice, North Mecha

## Abstract

**Objective:**

The aim of the study was to assess knowledge, attitude and preventive practice towards tuberculosis.

**Result:**

More than half of the study participants stated that bacteria are responsible agents in causing tuberculosis and as the same time 12.2% study participants thought that tuberculosis is not preventable disease. Overall, 54% of study participants had good knowledge, 68% had good attitude but only 48% had good practice in preventing tuberculosis. Compared to many national and international studies, knowledge, attitude and preventive practice towards tuberculosis was not satisfactory. Strengthening of awareness creation and health education program towards tuberculosis is needed.

## Introduction

Globally, 9.7 million people get sick with tuberculosis (TB) and 1.7 million people die from it, each year [1]. TB continues to be a major public health problem across the world, including Ethiopia. It causes ill-health among millions of people each year and ranks as the second leading cause of death from an infectious disease [2].

In 2006, 1.7 million deaths resulted from TB: the majority situated in sub-Sahara Africa [3]. Even though the incidence of TB has decreased worldwide, an estimated 10.4 million people developed TB in the year 2015 of which one-quarter was from Africa [4].

In every second; around the globe a person is infected with TB and every 10 s someone dies as a consequence of the disease [5].The 2018 Global TB report showed that Ethiopia is included in the 30 high TB burden countries [6] and in Ethiopia the case detection of the disease was 62 (51–74%) for all forms of TB [7, 8].

Raising communities’ awareness contributes for early diagnosis of TB which is one of the pillars of the End TB Strategy [4]. Studies documented a positive association between TB knowledge, care seeking and treatment adherence [9, 10]. To address such issues, the level of knowledge should be known to design an appropriate interventional programmes [11] in a specific regions.

The finding from various studies indicate that patient delay may be influenced by several factors, namely lack of knowledge, lack of awareness of the significance of symptoms, negative social attitudes or combinations of these [12].

Though few studies were conducted on knowledge, attitude and preventive practice (KAP) towards tuberculosis in Ethiopia, no study has been done about KAP towards tuberculosis among Mecha District communities. Therefore, this study was designed to investigate KAP of Mecha District communities towards tuberculosis.

## Main text

### Study design

A cross-sectional descriptive study was conducted on North Mecha district residents who visited the adult outpatient departments (OPD) of public health facilities for various medical, maternity and family planning services from April 901 to May 30/2018. The district has 290,546 total population which is located in West Gojjam Zone which is part of Amhara Region, Ethiopia. The district is situated at 530 km North West of Addis Ababa, capital city of Ethiopia. Merawi is the administrative city of the district [13]. In this district there are a total of 10 health centers and one primary Hospital with 96% TB cure rate, 97% TB success rate, and 85% Bacillus Calmette–Guérin (BCG) vaccination coverage. The Catchment area had a total population of 14,034 in the selected health institutions [14].

#### Sample size determination and sampling procedure

Sample size was calculated using; 95% confidence interval (CI), 5% margin of error (d), P as 45.9% taken from a study done in Gambella Region, Ethiopia [18]. Based on the above assumptions and by adding 10% non-response rate to the initial sample size, the sample size was 420.

From the total 11 health facilities (HFs) found in North Mecha district, 4 HFs (Wotet Abay Health center, Abyot Fana Health Center, Felege Birehan Health Center and Merawi Primary Hospital) were selected using simple random sampling method. Then based on the client flow, the total sample size was proportionally allocated to each selected HFs. Finally clients visiting each of the selected health facilities in the data collection period were selected using systematic random sampling technique.

The data collection questionnaire was developed after reviewing different relevant literatures. The questionnaire, first developed in English language and then translated to Amharic (local language). The questionnaire had four different parts. Part-I: comprising of socio-demographic questions, Part-II: comprising of twelve different knowledge assessing questions, Part-III: comprising of seven different attitude assessing questions and Part-IV: comprising of seven questions assessing the preventive practice towards TB.

Pretest was done on 5% of the total sample size at Meshenti Health Center. After the pretest, necessary modifications and correction took place to ensure validity. Four data collectors and one supervisor were recruited and trained for 1 day to collect and supervise the data respectively.

#### Data processing and analysis

Those respondents who scored greater than or equal to the mean value of knowledge questions were regarded as having good knowledge. Respondents who scored greater than or equal to the mean value of attitude assessing questions were regarded as having favorable attitude. The respondents’ score greater than or equal to the mean value of preventive practice assessing questions were regarded as a participant having good practice.

The data were checked and cleaned, coded using non-overlapping numerical codes. Computer data files were thoroughly checked for errors, implausible values and inconsistencies that might be due to coding, entry, typing and other errors. Then, it was exported to SPSS version 20 for analysis. Descriptive statistics, like percentage, mean and standard deviation was used for the presentation. Then the data were presented by using sentences, graphs, tables, frequencies, percentages.

### Result

Out of the total 420 sample size, 403 individuals participated in the study making the response rate 95.9%. Of which, 53.3% respondents were females. All the respondents’ fall in the age range between 12 and 82 years old, with 35 years being the mean age. Majority of respondents were Orthodox Christian religion followers and ethnically almost all (97.8%) were from Amhara ethnicity. The minimum monthly income of the study participants was no income to 25, 141 Ethiopian Birr (ETB) the maximum monthly income and 1, 124 ETB was the average monthly income (Table 1).

**Table 1:** Summary of socio-demographic distribution and knowledge, attitude and preventive practice towards TB, Mecha District, Northwest Ethiopia, 2018

Variables	Category	Knowledge				Attitude				Practice				Total	
		Good knowledge		Poor knowledge		Favorable attitude		Unfavorable attitude		Good practice		Poor practice			
		n	%	n	%	n	%	n	%	n	%	n	%	n	%
Age	< 21	35	67.3	17	32.7	43	82.7	9	17.3	39	75.0	13	25.0	52	12.9
	21–30	97	64.7	53	35.3	103	68.7	47	31.3	87	58.0	63	42.0	150	37.2
	31–40	47	56.0	37	44.0	53	63.1	31	36.9	38	45.2	46	54.8	84	20.8
	41–50	37	61.7	23	38.3	41	68.3	19	31.7	31	51.7	29	48.3	60	14.9
	51–60	17	43.6	22	56.4	23	59.0	16	41.0	15	38.5	24	61.5	39	9.7
	> 60	5	2.8	13	72.2	7	38.9	11	61.1	6	33.3	12	66.7	18	4.5
Marital status	Single	65	60.7	42	39.3	53	49.5	54	50.5	47	43.9	60	56.1	107	26.6
	Married	113	41.4	160	58.6	157	57.5	116	42.5	179	65.6	94	34.4	273	67.7
	Divorced	7	41.2	10	58.8	11	61.1	6	35.3	9	52.9	8	47.1	17	4.2
	Widowed	1	16.7	5	83.3	4	66.7	2	33.3	3	50.0	3	50.0	6	1.5
Educational status	Cannot read and write	31	24.6	95	75.4	49	38.9	77	61.1	55	43.7	71	56.3	126	31.3
	Can read and write	185	66.8	92	33.2	193	69.7	84	30.3	169	61.0	108	39.0	277	68.7
Religion	Orthodox	189	51.6	177	48.4	251	68.6	115	31.4	198	54.1	168	45.9	366	90.8
	Muslim	12	52.2	11	47.8	14	60.9	9	39.1	10	43.5	13	56.5	23	5.7
	Protestant	9	64.3	5	35.7	8	57.1	6	42.9	10	71.4	4	28.6	14	3.5
Ethnicity	Amhara	213	54.1	181	45.9	255	64.7	139	35.3	157	39.8	237	60.2	394	97.8
	Others^a^	4	44.4	5	55.6	6	66.7	3	33.3	3	33.3	6	66.7	9	2.2
Occupation	Government employee	192	86.5	30	13.5	155	69.8	67	30.2	181	81.5	41	18.5	222	55.1
	Farmer	11	12.2	79	87.8	28	31.1	62	68.9	31	34.4	59	65.6	90	11.7
	Students	57	70.4	24	29.6	62	76.5	19	23.5	43	53.1	38	46.9	81	15.1
	Others^b^	3	30.0	7	70.0	6	60.0	4	40.0	6	60.0	4	40.0	10	9.9
Monthly income (ETB)	< 1000	60	27.9	155	72.1	121	56.3	94	43.7	81	37.7	134	62.3	215	53.3
	1000–3000	98	58.3	70	41.7	111	66.1	57	33.9	72	42.9	96	57.1	168	41.7
	> 3000	12	60.0	8	40.0	14	70.0	6	30.0	9	45.0	11	55.0	20	5

^a^Tigre, Agew, Oromo

^b^Daily laborers, unemployed

From the total 403 study participants, 354 (87.8%) heard about tuberculosis. From these, majority (56.8%) of them received the information from health workers and followed by 26.8% from different Medias.

### Knowledge towards TB

From those study participants who had information about TB, 74% mentioned that droplet inhalation as the main mode of transmission of the disease whereas 2% replied that heredity as the mode of transmission of the disease. Regarding sign and symptoms of TB, 39.4% mentioned that cough for greater than or equal 2 weeks is the clinical manifestation of a client with TB. Forty-nine (12.2%) study participants thought that TB is not preventable disease. More than half (56%) of the study participants stated that bacteria is the responsible agent in causing TB.

#### Attitude towards TB

Regarding to communities attitude towards tuberculosis, 40.7% of study participants stated that TB is dangerous and serious for the community. Majority of study participants (46.2%) stated that TB is cannot transmitted from human to human. Almost one-fifth (19.3%) of study participants stated that discriminating TB patient is necessary.

#### Practice towards in the prevention of TB

Three hundred sixty-five (90.6%) of study participants’ house had window but only 60% of them open the window regularly. Almost one-fifth (19.4%) of study participants ever screened for TB. Two-third of the study participants ever received health education about TB. From all study participants, two-third stated that they will cover their mouth during coughing if they had TB as a measure to prevent further spread of the disease (Table 2).

**Table 2:** Communities’ practice towards for the prevention of Tuberculosis, Mecha District, Northwest Ethiopia, 2018

Variables	Value	n	%
Does your house has window?	Yes	365	90.6
	No	38	9.4
Do you open your home window regularly	Yes	219	60.0
	No	146	40.0
Do you open car window’s during travelling	Yes	118	29.3
	No	285	70.7
Have you ever screened for TB?	Yes	78	19.4
	No	325	80.6
Have you ever got health education about TB	Yes	268	66.5
	No	135	33.5
If you have TB, what do you do?	Consult health worker	347	86.1
	Consult traditional healers	43	10.7
	Kept silent	13	3.2
If you have TB, what measures would you do for the family, community	Cover my mouth and nose during coughing and sneezing	268	66.5
	I will cough arbitrarily	48	11.9
	I do not know	87	21.6

### Overall KAP level

**Fig. 1:**
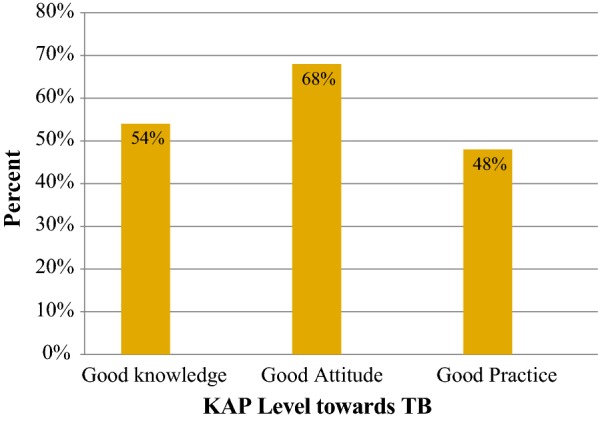
Study participants overall KAP level towards tuberculosis, Mecha District, Northwest Ethiopia, 2018

Two hundred eighteen (54%) of study participants had good knowledge towards TB and 193 (48%) of participants had good preventive practice in preventing TB (Fig. 1).

### Discussion

This study offers information on the knowledge, self-reported attitudes and practices towards TB. This finding entails that there is a significant gaps in preventive practice towards TB infection control.

It is a well-known fact that knowledge can influence people’s practices regarding prevention [15]. The current study revealed that the overall knowledge about TB was 54% which was lower than a study done in Iran [16] in which 62% of participants had good knowledge about TB. Whereas the current finding was better than a study done in Thailand in which 74.2% respondents had low level of knowledge about TB [17].

In the current study, 87.8% heard about tuberculosis which was lower from other studies conducted in different settings. Study done in Shinile town revealed that 94.9% heard about TB [18]. Another study done in middle and lower Awash valley of Afar region, Ethiopia [19] showed that, 92.8% and 95.6% of the study participants were aware of the disease, respectively. A study done in Libya [20], Sabah [12] and Iraq [21] also showed that 95%, 96% and 91% of the respondents heard about TB respectively. The discrepancies might be explained; study participants in the current study were from the rural communities whereas other studies were included communities from town/urban sites in which information access towards TB will be gain from health workers and Medias. But in the current study, majority of them responded the sign and symptoms of TB in a good way which was consistent with studies done in southwest Ethiopia [22], northeast Ethiopia [19], Iran [23] and Philippines [24].

The current study showed that the overall attitude towards TB was 68% which is better than a study conducted in Thailand [17] showed that 47.9% of study participants were categorized as they had high level of attitude.

The current finding indicated that the overall preventive practice towards TB was 48%. The finding was better than a study done in Iran [16] in which the overall preventive practices towards TB was 42.6%. Whereas a study done in Thailand [17] showed that 55.5% of study participants had high level of preventive behavior. The discrepancy might be due to that, those study participants from different countries may have differences in health education program design, health literacy, and access to health workers and Medias.

Regarding the screening practice for TB in the current study, 19.4% of study participants ever screened for TB which is consistent with a study conducted in Thailand [25] in which 18.6% of participants stated that they had undergone a TB screening test.

In regard to preventive measures, 66.5% of participants had practice of covering their mouth during coughing which is better than a nationwide study done in Mongolia [1] 42.9% of participants pointed covering their mouth and nose when coughing and sneezing. This difference might be the study done in Mongolia is a nationwide study in which more rural residents had a chance to be included and this may raise the chance of getting study participants with limited access to health information and practice.

Concerning the consultation practice, 86.1% of study participants in the current study stated that they would find a health worker if they got TB. This is lower than a study done in Sabah [12] in which 98% of respondents said that they would consult a doctor immediately.

### Conclusion

Compared to many national and international studies, the knowledge, attitude and preventive practice towards TB was not satisfactory. The finding from this research showed that majority of participants received information about the disease but their practical knowledge, attitude and practice was not as such comparative with the information they received. This urges the healthcare providers and other concerned bodies to design strategies to provide a better awareness creation towards the disease.

### Recommendation

Based on the gaps identified, this research forwards some recommendation to the concerned body.

Mecha District Health office: to strengthen the awareness creation and health education program towards TB in each healthcare facilities.

Other responsible stakeholders and NGOs: to launch awareness creation session about the impact of poor TB Practice for their health and the community.

## Limitations

The study was done on patients rather than community members using cross sectional study design in a single district. Study participants were not given the chance to respond for qualitative type questions so that we might not include their deep insight about their attitude and practices.

The study addresses only clients who came to health facilities.

## Data Availability

Not applicable.

## Abbreviations

BCG: Bacillus Calmette–Guérin
DR-TB: drug resistant tuberculosis
ETB: Ethiopian Birr
HFs: health facilities
HIV: human immune virus
KAP: knowledge, attitude and practice
MDR-TB: multidrug resistant tuberculosis
OPD: outpatient department
TB: tuberculosis
